# Closely related gull species show contrasting foraging strategies in an urban environment

**DOI:** 10.1038/s41598-021-02821-y

**Published:** 2021-12-08

**Authors:** K. A. Lato, D. J. Madigan, R. R. Veit, L. H. Thorne

**Affiliations:** 1 School of Marine and Atmospheric Sciences, Stony Brook University, Stony Brook, NY USA; 2 Department of Integrative Biology, University of Windsor, Windsor, ON Canada; 3 Department of Biology, College of Staten Island (CSI), CUNY, Staten Island, NY USA

**Keywords:** Stable isotope analysis, Urban ecology, Ecology

## Abstract

The expansion of urban landscapes has both negative and positive effects on wildlife. Understanding how different species respond to urbanization is key to assessing how urban landscapes influence regional wildlife behavior and ecosystem structure. Gulls are often described as strong urban adapters, but few studies have explored species-specific differences in habitat use. Here, we use GPS tracking in conjunction with stable isotope analysis (SIA) to quantify the habitat use and trophic ecology of great black-backed gulls (*Larus marinus*) and herring gulls (*L. argentatus*) in an urbanized area. Non-Metric Multidimensional Scaling (NMDS) of foraging locations revealed significant differences in the habitat use between species. Great black-backed gulls foraged primarily in marine habitats and herring gulls foraged primarily in specific urban habitats (e.g., landfills, dumpsters) and showed higher site fidelity in terms of the proportion of foraging sites revisited. Further, great black-backed gulls had significantly higher δ^15^N and δ^13^C than herring gulls, reflecting the use of marine, rather than urban, food sources. This study highlights the variability in urban habitat utilization among closely related species, assesses stable isotope signatures of urban diets in wild birds, and discusses ecological implications of the relative contribution of urban and marine foraging.

## Introduction

Urban landscapes have profound impacts on animal movement and behavior by affecting habitat structure and resource availability^1,2^. Urbanization is often associated with habitat fragmentation and high levels of noise and light pollution, which can result in avoidance behavior in native wildlife^3–5^. However, urban landscapes can also provide benefits to species that are more adaptable to such environments. For example, many animals can thrive in urban parks, nest on human-made structures, and exploit human refuse as a predictable and abundant food source^6–8^. This variability in urban adaptability among wildlife is a primary driver for reduced species richness and diversity associated with urban and suburban habitats^2,9^.

Animal movements and foraging behavior in response to urban landscapes can have further impacts on regional trophic dynamics and food web structure^10^. For example, when predators shift from foraging on natural prey to anthropogenic food, predation pressure and top-down forcing on lower trophic levels can be reduced^11,12^. This decoupling of predator–prey interactions could have broader ecosystem and food web effects, potentially increasing prey populations due to reduced predation or shifting the ecological niche space among congener predators over long timescales. Foraging and movement ecology of predatory urban adapters has recently gained attention^6,13,14^ while less emphasis has been placed on studying animals that may utilize urban environments to a lesser extent. Understanding how different species respond to urbanization is key to gaining a more holistic picture of the impacts of urban landscapes on wildlife and ecosystem structure. Thus, it is important to capture the wider breadth of responses to urbanization to understand, predict, and manage the impacts of urbanization on wildlife.

Gulls (*Larus* spp.) are ideal species to study the effects of urban landscapes on wildlife due to their highly plastic behavior, generalist feeding habits, and broad geographic distributions^15–18^. Multiple gull species have been documented nesting on rooftops, feeding at landfills, and even adjusting the timing of foraging activity based on human behavior^16,19,20^. While gulls are broadly generalized as strong urban adapters, the degree to which urban habitats are utilized likely varies across gull species despite close evolutionary relatedness and sympatric nesting habits^16^. This provides the opportunity to more closely investigate how urban habitats may influence animal movements and trophic ecology across species.

**Figure 1:**
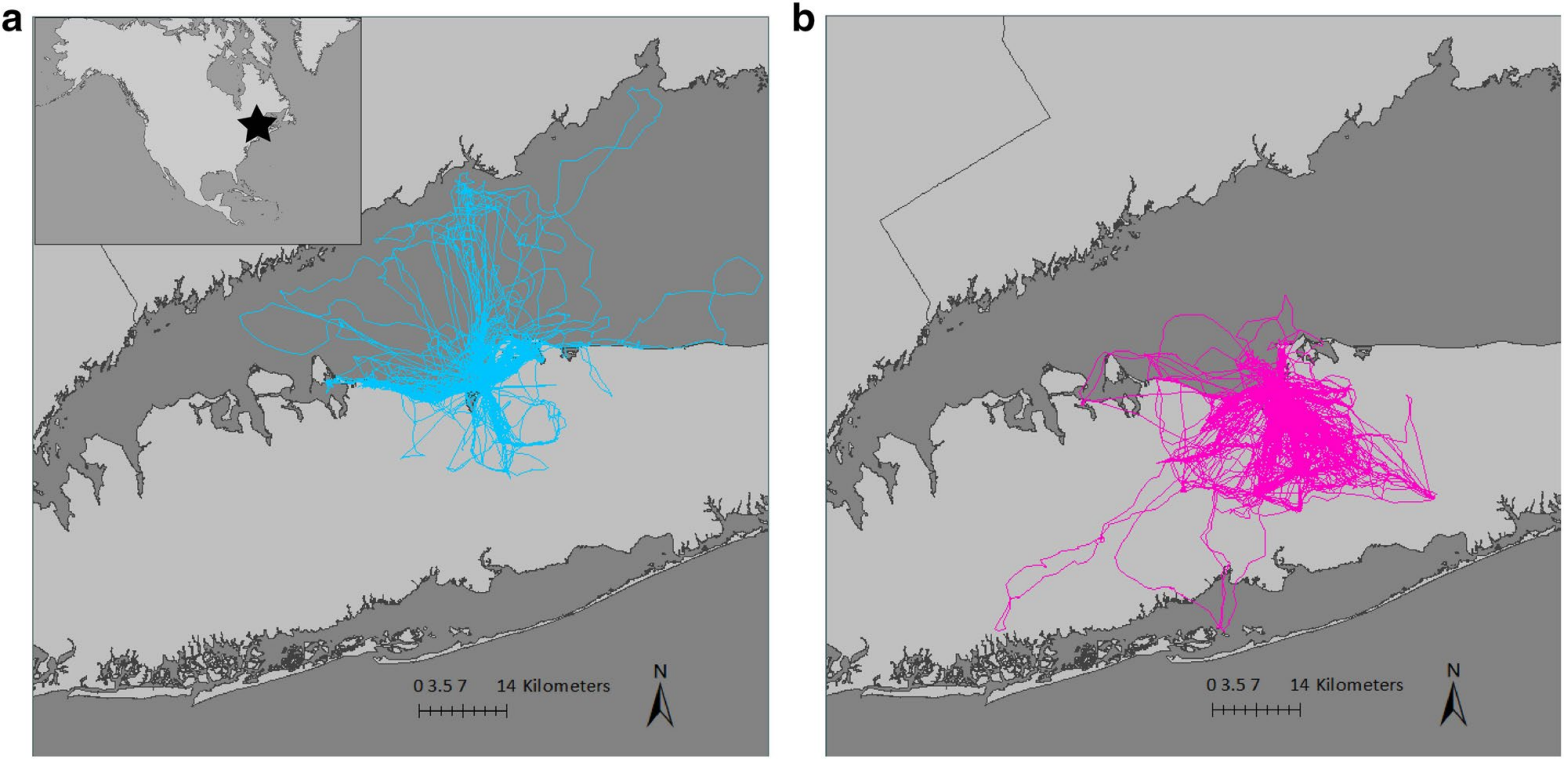
(**a**) GPS tracks of gulls from our study site showing great black-backed gulls (blue) traveling to open water and coastal areas on foraging trips and (**b**) herring gulls (pink) traveling toward urban habitats on foraging trips. The location of the study site on Long Island, NY in North America is shown (star symbol, inset). Maps produced using ArcGIS (v. 10.8.1).

Herring gulls (*Larus argentatus*) and great black-backed gulls (*L. marinus*) are considered generalist predators that feed in marine and urban environments^17,21–24^, but the relative importance of these foraging habitats to the two species is unclear. We investigated the habitat use of both species in an urban setting on Long Island, New York in 2019–2021 (Fig. 1). Using GPS tracking and stable isotope analysis we assessed how the habitat use, foraging behavior, and trophic ecology differs in herring and great black-backed gulls breeding in an urban environment.

## Results

### Habitat use and foraging behavior

GPS loggers were deployed on 27 great black-backed gulls and 31 herring gulls during egg incubation in 2019–2021. We recovered GPS data from 16 great black-backed and 25 herring gulls (Table 1), totaling 574 foraging trips and 9777 hours of tracking data. Tag deployments ranged from 5 to 22 days with an average tag duration of 9.7 days (± 5.1; Supplementary Table S1). Great black-backed and herring gulls showed distinct differences in habitat use (Fig. 1). Great black-backed gulls predominately used marine areas, including the Long Island Sound, on foraging trips. In contrast, herring gulls predominately used urban habitats and rarely spent time in marine habitats on foraging trips (Fig. 1).

**Table 1:** Summary of samples sizes and trip metrics with associated p-values from statistical comparisons of GPS tracking data for herring gulls (HERG) and great black-backed gulls (GBBG). Differences in trip metrics between species were tested using a Wilcoxon-rank sum test. Significant differences are shown in bold (α ≤ 0.05).

	HERG	GBBG	p-value
**Sample size (n)**			
GPS tracks	25	16	
Whole blood samples	40	44	
**Trip metrics**			
Trip duration (min)	136.00 ± 53.06	111.05 ± 76.7	**1.7 × 10**^**–2**^
Total trips distance (km)	22.65 ± 9.20	23.61 ± 11.72	8.0 × 10^–2^
Prop. of foraging sites revisited	0.46 ± 0.14	0.28 ± 0.15	**6.2 × 10**^**–3**^
Prop. of foraging trips revisiting same site	0.92 ± 0.07	0.85 ± 0.16	3.8 × 10^–1^

**Figure 2:**
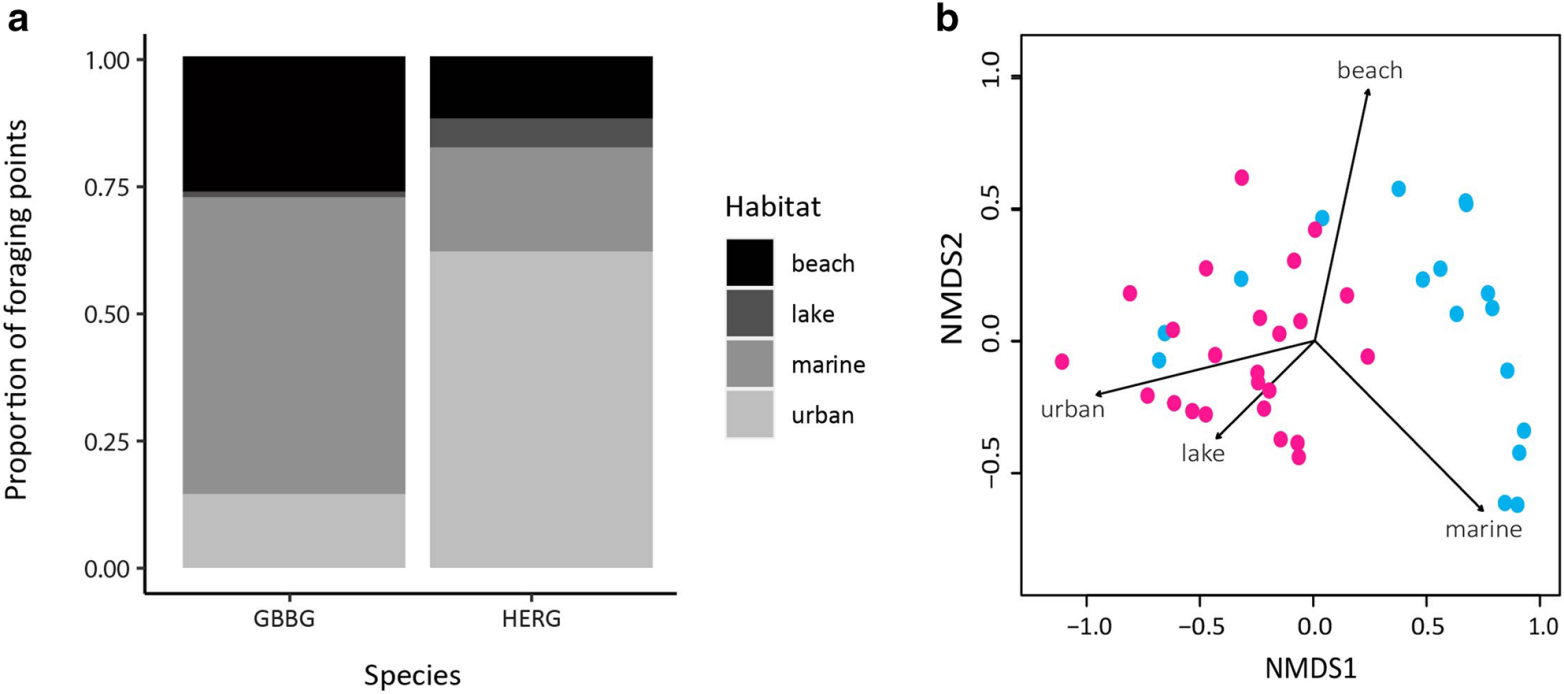
(**a**) Proportion of GPS foraging points for herring (HERG) and great black-backed gulls (GBBG) in different habitat types. Proportional value means of all tracked individuals. (**b**) Biplot of NMDS model for arcsine transformed proportions of foraging points in each habitat type with herring gulls in pink and great black-backed gulls in blue.

The Non-Metric Multidimensional Scaling (NDMS) analysis showed an excellent fit with the data (stress = 0.023) and highlighted differences in foraging habitats used by great black-backed and herring gulls. Habitat use of the two species was significantly different based on the mean ranked dissimilarities of proportions of habitat types used (ANOSIM R-statistic = 0.519, p = 1.0 × 10^–3^). Great black-backed gulls foraged more in marine and beach habitats and herring gulls utilized urban habitats to a greater degree than any other habitat type (Fig. 2). While there was no significant difference in total foraging trip distance between species (p = 8.0 × 10^–2^), great black-backed gulls had significantly shorter trip durations (p = 1.7 × 10^–2^; Table 1). Herring gulls revisited a significantly greater number of foraging sites than great black-backed gulls (p = 6.2 × 10^–3^) but there was no significant difference in the proportion of trips revisiting the same foraging site between species (p = 3.8 × 10^–1^; Table 1).

### Stable isotope analysis

**Figure 3:**
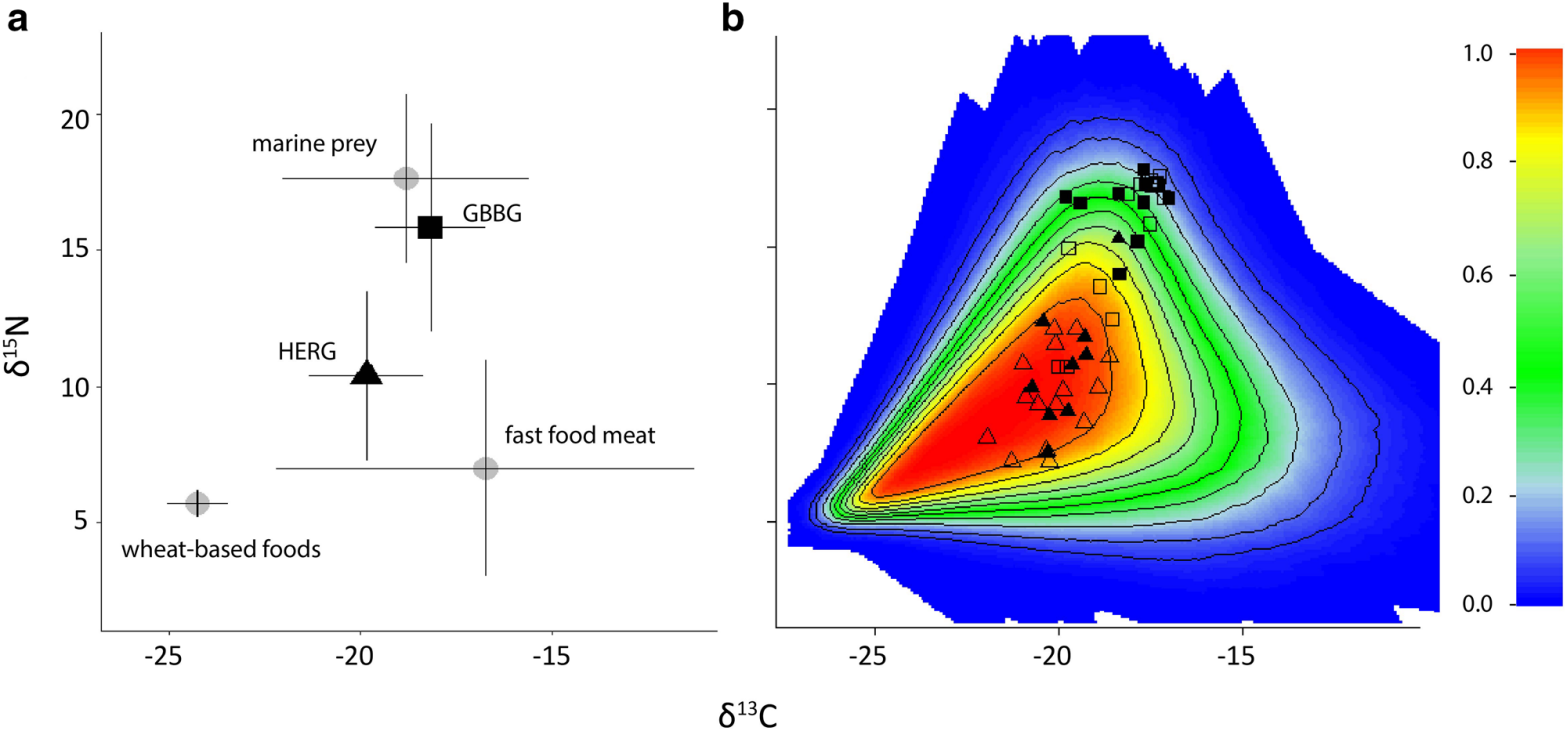
(**a**) Biplot of whole blood δ^15^N and δ^13^C for great black-backed gulls (black squares) and herring gulls (black triangles) and potential food sources (grey circles). Differences in δ^13^C and δ^15^N between great back-backed and herring gulls were tested for significance using a Wilcoxon-rank sum test (α = 0.05). (**b**) Simulated mixing region from potential food sources and probability contours. Isotopic positions of gulls with corresponding GPS tracks are shown as filled symbols (squares for great black-backed and triangles for herring gulls), and those without GPS tracks as open symbols. Contour lines are placed at 10% intervals except for the outermost contour, which is at the 5% level.

Whole blood samples were collected from 44 great black-backed and 40 herring gulls (Table 1; Supplementary Table S2). Great black-backed gulls had significantly higher δ^15^N and δ^13^C values than herring gulls (Fig. 3a; Wilcoxon rank-sum p = 6.8 × 10^–14^; p = 9.6 × 10^–15^). Marine prey (crabs, fish and bivalves; see Supplementary Table S3 for species and SI values) showed higher δ^15^N values than urban sources of food. Fast-food meat, observed in the diet of urban-foraging birds, showed a wide range of δ^13^C values that were higher than those in marine prey items (− 16.1 ± 2.7‰), while wheat-based items showed lower δ^13^C values than marine prey (− 25.3 ± 0.4‰; Fig. 3a; Supplementary Table S3).

**Figure 4:**
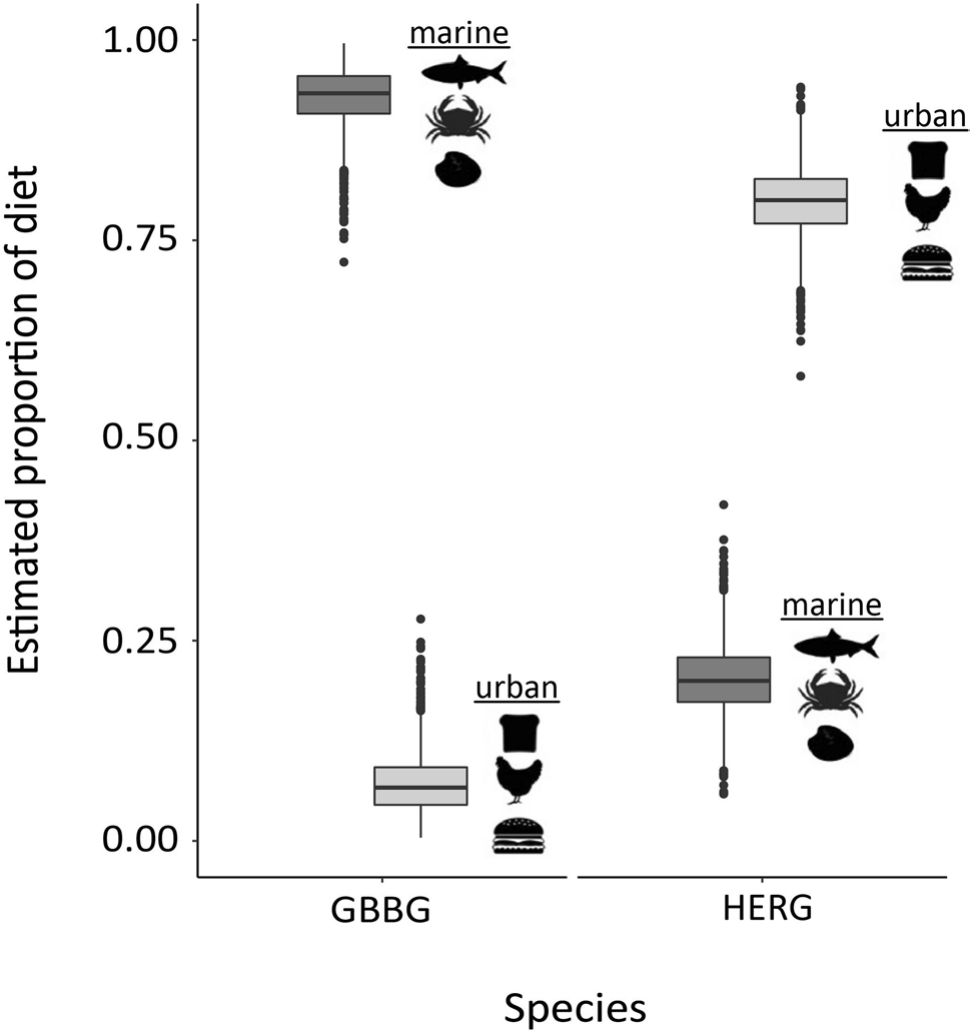
Estimated proportions of urban food (light grey) and marine prey (dark grey) in the diets of great black-backed gulls and herring gulls, respectively. Diet proportions were estimated using Bayesian isotopic mixing model (MixSIAR^26^) using δ^15^N and δ^13^C values of gull whole blood and gull diet-based urban food (bread, fast-food meat) and marine food (bivalves, crabs, fish) sources.

Simulated mixing polygons showed that great black-backed and herring gull δ^15^N and δ^13^C values fell within the isotope mixing model’s isotopic mixing space (i.e., the outermost 5% contour of polygons) and thus were considered well represented by the diet items sampled^25^ (Fig. 3b). Posterior density outputs from our isotope mixing model showed higher diet contributions of marine diet sources (92 ± 4%) than urban sources (8 ± 4%) in great black-backed gulls (Fig. 4). In contrast, herring gull diet estimates showed higher urban contributions (80 ± 4%) than marine contributions (20 ± 4%) (Fig. 4). While both species exhibited strong preferences in feeding habits on the population level, individual mixing model estimates suggested a more generalist diet in herring gulls, further demonstrated by higher variability of δ^13^C values (Fig. 3a).

## Discussion

We found marked differences in both the spatial habitat use and diet of two co-nesting, closely related *Larus* species in an urban environment, reflecting species-specific differences in urban vs. marine habitat use. Herring gulls primarily foraged in urban habitats and revisited foraging sites more frequently than great black-backed gulls, which primarily foraged in marine areas. Our stable isotope mixing model showed that herring gulls primarily consumed urban food items (wheat, fast-food meat) and great black-backed gulls primarily consumed marine prey (bivalves, crabs, fish). We observed individual specialization in foraging great black-backed gulls, with 4 individuals tracked in 2021 and 2 blood samples from non-tracked individuals in 2020 reflecting a high degree of urban foraging in contrast to our other observations of this species in this study.

Herring gulls exhibited higher site fidelity in terms of the proportion of foraging sites revisited across trips, which is likely due to the high spatial and temporal predictability of urban food sources such as dumpsters and landfills^17,18,27,28^, further exemplified by several individual herring gulls that showed similar flight paths and regularly visited foraging sites in the same order on each trip. The predictability of foraging areas could have important energetic and reproductive implications, as birds foraging on predictable food sources likely spend less time searching for food and show more directional travel than birds foraging on ephemeral natural prey patches. Conversely, it is possible that marine prey, such as fish, may provide a greater nutritional benefit than urban refuse^29^ and thus may be seen as a preferred food resource in the case of great black-backed gulls. Though we found no significant difference in the proportion of trips revisiting the same foraging sites between species, this metric for great black-backed gulls was dominated by birds tracked in 2021 which traveled frequently to a large coastal harbor (Port Jefferson Harbor) whereas birds from 2019 and 2020 utilized a greater variety of marine areas (e.g., Long Island Sound). This highlights the importance of not only individual specialization but also interannual variability in tracking studies. Total trip distance did not significantly differ between herring and black-backed gulls, but for urban feeding gulls this metric is likely a reflection of proximity to nearby urban food sources rather than search effort or energetic expenditure (e.g., birds feeding in predictable urban environments will only travel as far as the landfill or urban site). Together our findings suggest that the foraging habits of herring gulls are highly dependent on the abundance and distribution of human sources of food. This notion is supported by other studies that have found urban gulls shift the timing of foraging based on anthropogenic activities when food resources become locally abundant^20^. This reliance of herring gulls on urban food resources could make them more vulnerable to changes in human behavior in this region than great-black backed gulls, which in contrast may be more influenced by changes in the natural environment (i.e. natural prey abundance). Over the last few decades, population expansion and fluctuation of herring gulls in the northeast United States has been attributed to changes in waste management practices, with populations increasing with landfill availability and declining after landfill closures^30,31^. However, species-specific patterns of reliance on urban food sources may vary regionally. For example, in the 1990s, great black-backed gulls off Nantucket, Massachusetts were more closely tied to feeding on the Nantucket landfill than herring gulls. Thus, great black-backed gulls were differentially impacted by the closure of the landfill in the early 1990s and experienced almost complete reproductive failure (R.R. Veit, pers comm). Previous studies have highlighted the impacts of waste management practices on urban gull populations, with reduced reproduction and breeding colony size following reduced access to urban food^32–34^. Differences in the diet and foraging behavior of gull species and their reliance on urban sources of food could have important implications for their population growth and stability as urban environments continue to expand in many regions of the globe.

Our results exemplify the variability in urban habitat use that exists among closely related species and is consistent with the few other studies that have examined this response across other closely related species. For example, red foxes (*Vulpus vulpus*) are found to utilize urban environments to a greater extent than the gray fox (*Urocyon cinereoargenteus*)^14,35^ in North America and, similarly, the evening bat (*Nycticeius humeralis*) is more sensitive to urban development than the big brown bat (*Eptesicus fuscus*), as studied in Indiana, USA^36^. Understanding the variation in urban habitat use across similar species is key to assessing the impacts of urbanization on wildlife and informing conservation and management in urban areas. While gulls are generally thought of as strong urban adapters, within the genus *Larus* there are species that will likely benefit more from the expansion of urban landscapes than others.

We found significant differences in δ^13^C and δ^15^N values between herring gulls and great black-backed gulls, demonstrating that the relative proportions of urban and marine food within the diet can alter animals’ isotopic values. While together the δ^13^C and δ^15^N values of gull diets distinguished well between urban and marine diets, we found that urban foods likely eaten by gulls had a wide range of δ^13^C values; fast-food meat frequently showed δ^13^C values less negative than those in marine prey but wheat-based products consistently showed δ^13^C values more negative than those in marine prey. These findings suggest that anthropogenic foods have a wide range of δ^13^C values and thus the type of urban refuse consumed by gulls would impact their resulting δ^13^C values. Our results showed that wheat contributes more heavily to herring gulls’ diets than fast-foot meat, despite the pervasiveness of corn in agricultural production and fast-food practices^37,38^. This is important to note as previous studies using isotope mixing models to reconstruct gull diets have largely used corn-fed meat to represent human refuse^39,40^ which could, in turn, provide inaccurate interpretations of gull foraging patterns.

Our results support the notion that consumers that feed extensively on urban food items can show different trophic positions than those feeding in natural habitats, potentially decreasing predation pressure on prey populations. The use of urban food sources by abundant consumers such as gulls could thus have important and far-reaching impacts on the structure and dynamics of local food webs. It is important to note that population fluctuations of gulls and other urban adapters are thought to be closely tied to anthropogenic resources, and thus population expansion of some species in some regions may be the direct result of increased availability of human waste and fishery discards^15^, though this may not be true for all populations^41,42^. In our study system, reduced predatory pressure by herring gulls on local systems is unquantified, and may not be linearly related to gull population size. Further quantitative studies could be performed examining the niche space of gulls within urban and marine environments to better understand implications of urban and marine foraging on trophic dynamics.

This research was conducted while birds were incubating eggs, and the spatial and temporal constraints of breeding may influence foraging behavior, diet, and competition between species. Coastal birds are central place foragers during the breeding season and both males and females have to return to the nest site regularly to incubate their eggs and feed their chicks^43^, which limits foraging trips in space and time. Spatiotemporal constraints on foraging could drive resource partitioning (i.e. the division between species of a particular resource often driven by interspecific competition) and promote species coexistence^44,45^. Previous studies have suggested that sympatric nesting seabirds, including gulls, compete for various natural resources during the breeding season and often divide their foraging habitat and diet items^46–49^. If urban resources continue to be abundantly available, they may provide an important resource to help alleviate interspecific competition of seabird colonies during the breeding period. It is hypothesized that stark differences in foraging strategies observed between herring and great black-backed gulls are reduced outside of the breeding period after the cessation of central place foraging, though this is currently supported only by anecdotal evidence. Other factors including season, prey availability, breeding stage, and colony size could influence the proportion of urban foraging by gulls^22,50,51^.

The implications of feeding primarily on urban vs. natural prey resources on population dynamics of urban adapters are unclear. Though there is no strong consensus, some studies have found that individuals feeding on urban food items have higher reproductive success and greater body condition^52,53^, most likely due to the higher caloric density and abundance of urban food. However, other studies have found that reproductive success is lower for urban foragers, potentially due to overall low nutrient concentrations in urban food^54,55^. The implications of urban feeding for individual health and population dynamics likely depend on the nutritional quality of specific food items taken. Foraging on urban refuse may also have further implications for immune health by exposing urban adapters to additional disease, toxins, parasites, and microbial communities^17,56,57^. Further studies could elucidate the ecological tradeoffs of urban foraging and to distinguish short-term (e.g., energetic) and long-term (e.g., individual health and longevity) impacts of urban foraging.

## Methods

### Study site and sample collection

Great black-backed and herring gulls were studied at Young’s Island (Fig. 1), a small island ~ 0.2 km from the coast in Stony Brook, Long Island, NY. Approximately 300 pairs of herring gulls and 100 pairs of great black-backed gulls nest on Young’s Island along with several other shorebirds and seabirds including great egrets (*Ardea alba*), least terns (*Sternula antillarum*), and American oystercatchers (*Haematopus palliatus*). Animals were observed, tagged, and sampled during the incubation period of the breeding season in 2019–2021.

Gulls were captured using bownet traps and noose carpets. Foraging movements of gulls were assessed by attaching CatLog Generation 2 (Catnip Technologies, Hong Kong) and igotU GT-120 (Mobile Action Technologies, Taiwan) GPS loggers to the central 3–4 tail feathers using Tesa Tape (Tesa Tape, Inc., NC), with each device equal to 1–2% of total gull body mass. GPS devices were programmed to take positions at 2-min intervals and lasted 5–22 days after deployment. Birds were recaptured using the same methods described above to recover GPS tags and collect blood samples. Upon tag recovery, blood samples were taken from the medial metatarsal vein using a 25-gauge needle and 3.0 mL syringe. We collected additional blood samples from randomly sampled individuals at the colony to increase sample size for isotopic analysis. Blood samples were immediately transferred to a 4.0 mL sodium heparin coated vacutainer and placed on ice, and subsequently transferred to a − 80 °C freezer until further processing.

Gulls were observed feeding on and regurgitating both marine (e.g., fish, bivalves) and urban (e.g., bread, chicken) food sources at the colony. Most diet samples (hard clam (*Mercenaria mercenaria*), ribbed mussel (*Geukensia demissa*), Atlantic menhaden (*Brevoortia tyrannus*), lady crab (*Ovalipes ocellatus*), spider crab (*Libinia emarginata*), horseshoe crab (*Limulus polyphemus*)) were collected opportunistically either from gull regurgitations during handling or during observations at the colony. Observations of foraging gulls suggested that gulls were also feeding on scup (*Stenotomus chrysops*), so samples of scup were collected from local fisherman. Additional urban diet items (hamburger, chicken finger, white bread) were collected from local fast-food restaurants following observations of gulls feeding on these items within urban habitats.

### Foraging movement analysis

Gulls often make short movements around the colony assumably to rest and drink, thus only foraging trips that were at least 20 min in duration and 0.5 km away from the colony were considered in our analysis. Additionally, we only analyzed birds that took at least 6 foraging trips during tag deployment to ensure that tracks accurately represented individual gull foraging behavior and habitat use^58^. Area restricted search (ARS) behavior was identified using the first passage time (FPT) analysis, which is based on the time it takes an animal to travel through a spatial circle with a given radius^59^. FPT values were assigned to each GPS point along a track line at varying radii sizes (10–250 m), and the radius at which the maximum variance in the log of FPT values occurred was determined to be the radius at which the animal is foraging. After FPT values were calculated for each point at the associated foraging radius, we adjusted for spatial autocorrelation according to Suryan et al.^60^. Here, each track line was subsampled by identifying the point of maximum FPT value and excluding all other points within a 2 × radius of search behavior, continuing this method until the entire track line was subsampled. If the search radius was < 30 m, we used a minimum buffer distance of 30 m of exclusion during adjustment for autocorrelation. Of those remaining subsampled points, the upper quartile of FPT values were considered the most intense search behavior and classified as foraging (ARS) points. When there were noticeable drift points within foraging points in our FPT analysis (n = 1 individual), likely representative of a bird drifting with strong tidal currents, only the first GPS point was considered foraging and all other drift points were excluded prior to further analysis. To account for any potential effect that trapping and handling may have on gull behavior, any initial foraging trips that were shorter in total trip distance compared to all subsequent trips was removed from further analysis.

Resulting foraging points were overlaid onto satellite imagery in ArcGIS (version 10.6.1) and categorized as either urban, marine, beach, or lake. ‘Beach’ was considered a separate category from ‘marine’, as birds feeding in this habitat could have been feeding on marine items or refuse from human beachgoers. To account for tidal range, GPS points within a 10 m buffer of the beachline were also classified as ‘beach’. Similarly, ‘lake’ was included as its own habitat as birds could have been feeding on fish or invertebrate prey or refuse from park visitors. We further quantified gull behavior using the following trip metrics: trip duration, total trip distance, and site fidelity. Site fidelity for each bird was assessed using two measurements that emphasize different aspects of fidelity: the proportion of foraging sites revisited reflected the extent of fidelity across foraging sites while the proportion of trips revisiting the same foraging site provided a metric of fidelity through time. In urban and lake habitats, foraging sites were defined by geographic boundaries (e.g., the geographic boundary of a strip mall). For GPS points in beach and marine habitats, foraging sites were defined by placing a 1.0 km buffer around each GPS point (e.g., if two GPS points were within 1.0 km of each other the bird was considered to visit that same site). This 1.0 km scale was based on the scale of habitat use within other (e.g urban and lake) habitats.

We assessed differences in the proportion of ARS points in each habitat type (urban, marine, beach, or lake) between great black-backed and herring gulls using Non-Metric Multidimensional Scaling (NMDS). We applied the arcsine transformation to proportions and calculated dissimilarities between individual gulls using Euclidean distances. NMDS is an ordination method that uses ranked distances to explore relationships between individuals or species and environmental space without assuming linear relationships between variables^61^. We assessed the fit of the NMDS using the stress value and the rule of thumb that stress values < 0.05 reflect an excellent fit, values < 0.1 reflect a good fit, values < 0.2 indicate an acceptable fit, and values > 0.3 reflect a poor fit^61^. We overlaid foraging habitat categories onto the NMDS plot to examine how habitat type related to observed patterns of habitat use in herring and great black-backed gulls. All trip metrics were assessed using Wilcoxon rank-sum tests. GPS tracking analyses were performed using the ‘adehabitatLT’ package in R and NMDS was performed using the ‘vegan’ package in R (R Core Team 2021, version 4.0.4).

### Stable isotope analysis

We analyzed gull whole blood for δ^13^C and δ^15^N values. The isotopic turnover rate in avian blood is approximately 2 weeks^62^ and thus reflects diet on a similar timescale as our GPS tracking data. Gull blood and diet samples were dried using either a freeze-dryer (FreeZone 6 Liter Benchtop Freeze Dry System Model Number: 553477; for 48 h) or a drying oven (Heratherm OGS400; 60 °C for 48 h). Dried samples were then homogenized to a fine powder using a porcelain mortar and pestle. Dried tissue sample was weighed out (0.3–0.6 mg) and encapsulated in tin capsules. For plant-based diet items (wheat), more sample mass (1.0–3.0 mg) was used due to lower plant N content and the consequential need for more sample material to obtain a δ^15^N measurement. δ^13^C and δ^15^N measurements were calculated as deviations from the standard in parts per thousand (‰), expressed in δ notation using the equation:

$$\updelta {\text{X }} = \, \left[ {\left( {{\text{R}}_{{{\text{sample}}}} {/}{\text{ R}}_{{{\text{standard}}}} } \right) \, {-} \, 1} \right] \, \times \, 1000$$

where X is ^13^C or ^15^N and R is the ratio of the heavier isotope to the lighter isotope (^15^N/^14^N or ^13^C/^12^C). δ^13^C and δ^15^N values were obtained using a Costech ECS 4010 Elemental Combustion System using a Zero Blank Autosampler, coupled to a ThermoFinnigan Delta Plus XP. Analytical precision was assessed using repeat sampling of the standard (tuna white muscle tissue; ± 0.2‰). Replicates of blood samples were also performed every tenth sample to further ensure precision of measurements (± 0.1‰).

We arithmetically corrected δ^13^C values for potential effects of lipids in gull whole blood, marine prey muscle tissue, and fast-food meat according to C/N ratios using the algorithm in Post et al.^63^ No lipid correction was performed for wheat diet samples as %C in wheat was ~ 40%, indicating negligible lipid content^63^. We chose to perform mathematical corrections for lipid content rather than chemical lipid extraction as chemical extraction can artificially alter δ^15^N values^64^, and we were not able to perform separate analyses for δ^13^C and δ^15^N across samples due to limited tissue material.

### Stable isotope mixing model

To estimate the relative contributions of marine and urban diet items to gull diet, we used the isotope mixing model MixSIAR in R (version 10.4). We chose this mixing model for its Bayesian framework, which allows the incorporation of elemental concentrations in diet items and uncertainty in trophic enrichment factors (TEFs)^26^.

We assessed the appropriateness of diet sources selected for the mixing model (described above, *Sample Collection*) using simulated mixing polygons^25^, which incorporates the standard deviation of dietary sources and source-specific TEFs, with 1500 iterations. The resulting output from this analysis represents the probability that a consumer’s diet is accurately captured in the proposed mixing model^25^. If an individual consumer falls outside this simulated mixing polygon space, the diet of that individual is considered to be poorly represented isotopically, and that sampling point removed from the isotope mixing model analysis.

TEFs (Δ^13^C and Δ^15^N) and associated standard deviations specific to consumer tissue (here, avian whole blood) and diet material (i.e., marine prey muscle, fast-food meat) were collected from literature, using different TEFs for different diet types due to well-defined relationships between TEFs and diet SI values^65^. For marine diet items, we used TEFs of − 0.3 ± 0.8 and 3.1 ± 0.2 for Δ^13^C and Δ^15^N, respectively^66^ (Hobson 1992). As there are no published TEFs for gull blood from a wheat-based or fast-food meat diet, we calculated TEFs for these diet items as diet-dependent discrimination factors according to Caut et al.^65^ Δ^13^C values for wheat-based items and fast-food meat were calculated to be 1.0 ± 0.4 and − 0.8 ± 0.4, respectively. Δ^15^N values for wheat-based items and fast-food meat were taken as the average TEF of avian blood (2.25 ± 0.2), as presented in Caut et al.^65^. The sensitivity of our results to variations in TEFs was assessed using values presented in additional literature for other avian species (Supplementary Table S4)^66,67^. We included gull species as a fixed effect and individual as a random effect in our model. We excluded year as a random effect, as model testing revealed that the inclusion of year did not improve our model according to the Akaike information criterion (AIC). Our model was run with 3 chains, a 500,000 burn-in period, and 1,000,000 iterations with a thinning rate of 500. Model convergence was assessed using the Gelman-Rubin and Geweke diagnostic tests. Diet items were grouped a priori based on foraging region (i.e., marine versus urban environments) and compositional (i.e., muscle, meat type) and isotopic similarity resulting in the following diet groupings: fast-food meat, wheat, and marine (Supplementary Table S3). Fast-food meat and wheat sources were combined a posteriori into an ‘urban food’ category.

### Declarations

The work in this study was approved by Stony Brook University’s Institutional Animal Care and Use Committee (IACUC #875550), conducted in accordance with relevant guidelines and regulations including ARRIVE (https://arriveguidelines.org) guidelines. GPS tag attachment was conducted under state (#2036) and federal (#22795) permits. Access to our study site was approved by the Department of Environmental Conservation (Temporary Revocable Permit #2019-002; 2020-01).

## Supplementary Information

Supplementary Tables.
