# Author Correction: Closely related gull species show contrasting foraging strategies in an urban environment

**DOI:** 10.1038/s41598-022-10158-3

**Published:** 2022-04-06

**Authors:** K. A. Lato, D. J. Madigan, R. R. Veit, L. H. Thorne

**Affiliations:** 1 School of Marine and Atmospheric Sciences, Stony Brook University, Stony Brook, NY USA; 2 Department of Integrative Biology, University of Windsor, Windsor, ON Canada; 3 Department of Biology, College of Staten Island (CSI), CUNY, Staten Island, NY USA

Correction to: *Scientific Reports* 10.1038/s41598-021-02821-y, published online 08 December 2021

The original version of this Article contained an error in the Method section, under the subheading ‘Stable isotope analysis’ where the formula was incorrect.

$$\updelta {\text{X}} = \left[ {\left( {{\text{R}}_{{{\text{sample}}}} - {\text{R}}_{{{\text{standard}}}} } \right) - 1} \right] \times 1000 $$

now reads:

$$\updelta {\text{X}} = \left[ {\left( {{\text{R}}_{{{\text{sample}}}} /{\text{R}}_{{{\text{standard}}}} } \right) - 1} \right] \times 1000 $$

The original Article has been corrected.

