# Supplementary material for: Closely related gull species show contrasting foraging strategies in an urban environment
Source: Sci Rep. 2021 Dec 8;11:23619. doi: 10.1038/s41598-021-02821-y (PMC8655058; doi:10.1038/s41598-021-02821-y)
Supplement: Supplementary file 1 — Supplementary Tables. [file 41598_2021_2821_MOESM1_ESM.docx]

Supplementary information for:

Closely related gull species show contrasting foraging strategies in an urban environment

Authors: KA Lato, DJ Madigan, RR Veit, LH Thorne

**Supplementary table S1**: The dates and durations of GPS tag deployments for all herring gulls (HERG) and great black-backed gulls (GBBG) included in the GPS tracking analysis.

| Bird ID | Species | Date Deployed (mm-dd-year) | Deployment Duration (days(hours)) |
| --- | --- | --- | --- |
| 304 | HERG | 05-22-2019 | 6 (144) |
| 305 | HERG | 05-24-2019 | 11 (265) |
| 313 | HERG | 05-22-2019 | 9.25 (222.07) |
| 406 | HERG | 05-24-2019 | 7.66 (184) |
| 456 | HERG | 05-24-2019 | 4.9 (118) |
| 633 | HERG | 05-18-2020 | 18.9 (454) |
| 635 | HERG | 05-18-2020 | 17 (417) |
| 642 | HERG | 05-18-2020 | 7.9 (189) |
| 641 | HERG | 05-20-2020 | 7.2(174) |
| 647 | HERG | 05-20-2020 | 7.1(170) |
| 648 | HERG | 05-20-2020 | 15(363) |
| 636 | HERG | 05-20-2020 | 5.9 (141) |
| 645 | HERG | 05-20-2020 | 19 (458) |
| 646 | HERG | 05-20-2020 | 5.7 (138) |
| 653 | HERG | 05-22-2020 | 8 (194) |
| 607 | HERG | 05-22-2020 | 7.7 (184.7) |
| 611 | HERG | 05-24-2020 | 13.2 (317.78) |
| 621 | HERG | 05-22-2020 | 7.7 (189) |
| 621 | HERG | 05-26-2021 | 6.72 (161.29) |
| 314 | HERG | 05-26-2021 | 24.90 (597.51) |
| 679 | HERG | 05-27-2021 | 8.07 (193.72) |
| 676 | HERG | 05-27-2021 | 8.03 (192.82) |
| 655 | HERG | 05-27-2021 | 7.91 (189.89) |
| 682 | HERG | 05-31-2021 | 7.20 (172.85) |
| 683 | HERG | 05-31-2021 | 7.77 (186.5) |
| 678 | HERG | 05-31-2021 | 7.83 (188.00) |
| 415 | GBBG | 04-26-2019 | 6.3 (152.2) |
| 404 | GBBG | 05-03-2019 | 4.7 (114) |
| 417 | GBBG | 05-08-2019 | 5.5 (133) |
| 403 | GBBG | 05-12-2019 | 12.2 (293.8) |
| 410 | GBBG | 05-12-2019 | 6.2 (148.5) |
| 414 | GBBG | 05-12-2019 | 6 (144) |
| 314 | GBBG | 05-03-2020 | 4.77 (114.5) |
| 400 | GBBG | 05-05-2020 | 5.65 (135.6) |
| 416 | GBBG | 05-05-2020 | 6.55 (157.23) |
| 610 | GBBG | 04-26-2021 | 19.25 (462.01) |
| 654 | GBBG | 04-26-2021 | 15.08 (361.98) |
| 664 | GBBG | 04-26-2021 | 8.02 (192.38) |
| 637 | GBBG | 04-26-2021 | 13.91 (333.76) |
| 668 | GBBG | 04-26-2021 | 5.83 (140.02) |
| 672 | GBBG | 04-26-2021 | 21.94 (526.55) |
| 639 | GBBG | 05-03-2021 | 6.80 (163.13) |

**Supplementary table S2**: All herring gull (HERG) and great black-backed gull (GBBG) blood samples included in stable isotope mixing models and their associated isotopic values. Sample numbers were assigned using the bird’s GPS tag ID number. Sample IDs ending in “M” represent the mate of a GPS tagged bird. Sample IDs beginning with “UNK” represent samples taken from birds that were not associated with a GPS-tagged nest.

| Bird ID | Species | Year | ẟ^13^C ‰ | ẟ^15^N ‰ |
| --- | --- | --- | --- | --- |
| 304 | HERG | 2019 | -20.5 | 7.8 |
| 305 | HERG | 2019 | -20.9 | 10.0 |
| 456 | HERG | 2019 | -20.6 | 12.2 |
| 302 | HERG | 2019 | -19.9 | 9.2 |
| 313M | HERG | 2019 | -18.7 | 15.0 |
| 607 | HERG | 2020 | -21.1 | 10.8 |
| 611 | HERG | 2020 | -20.5 | 7.9 |
| 621 | HERG | 2020 | -21.0 | 9.7 |
| 633 | HERG | 2020 | -20.7 | 9.5 |
| 635 | HERG | 2020 | -20.3 | 11.5 |
| 636 | HERG | 2020 | -22.0 | 8.4 |
| 641 | HERG | 2020 | -18.9 | 11.1 |
| 642 | HERG | 2020 | -20.4 | 7.6 |
| 645 | HERG | 2020 | -19.5 | 8.9 |
| 646 | HERG | 2020 | -20.1 | 9.9 |
| 647 | HERG | 2020 | -21.4 | 7.6 |
| 648 | HERG | 2020 | -19.7 | 12.05 |
| 651 | HERG | 2020 | -19.2 | 10.0 |
| 652 | HERG | 2020 | -20.3 | 12.0 |
| 653 | HERG | 2020 | -20.2 | 9.5 |
| UNK6 | HERG | 2020 | -19.5 | 11.1 |
| UNK7 | HERG | 2020 | -19.8 | 10.8 |
| UNK8 | HERG | 2020 | -20.4 | 9.1 |
| UNK9 | HERG | 2020 | -19.5 | 11.7 |
| 622 | HERG | 2021 | -19.7 | 9.6 |
| 652 | HERG | 2021 | -18.6 | 10.0 |
| 211698 | HERG | 2021 | -19.6 | 10.1 |
| 211701 | HERG | 2021 | -20.1 | 11.5 |
| 314 | HERG | 2021 | -18.6 | 10.4 |
| 676 | HERG | 2021 | -20.3 | 10.1 |
| 678 | HERG | 2021 | -20.2 | 11.4 |
| 682 | HERG | 2021 | -19.5 | 10.6 |
| 683 | HERG | 2021 | -19.4 | 13.1 |
| UNK7 | HERG | 2021 | -19.9 | 8.0 |
| UNK8 | HERG | 2021 | -20.3 | 10.4 |
| UNK9 | HERG | 2021 | -18.5 | 12.9 |
| UNK10 | HERG | 2021 | -19.5 | 10.5 |
| UNK11 | HERG | 2021 | -19.7 | 11.0 |
| UNK12 | HERG | 2021 | -19.5 | 9.3 |
| UNK14 | HERG | 2021 | -20.0 | 10.6 |
| 407M | GBBG | 2019 | -18.8 | 12.3 |
| 417 | GBBG | 2019 | -18.7 | 16.5 |
| 415M | GBBG | 2019 | -17.9 | 15.5 |
| 410M | GBBG | 2019 | -20.2 | 10.7 |
| 403M | GBBG | 2019 | -17.7 | 16.8 |
| 410 | GBBG | 2019 | -18.0 | 16.2 |
| 402 | GBBG | 2019 | -18.6 | 13.8 |
| 412 | GBBG | 2019 | -20.0 | 16.4 |
| 414 | GBBG | 2019 | -18.2 | 14.9 |
| 408 | GBBG | 2019 | -17.4 | 16.4 |
| 235 | GBBG | 2020 | -19.6 | 14.7 |
| 235M | GBBG | 2020 | -18.5 | 16.5 |
| 307 | GBBG | 2020 | -17.8 | 16.8 |
| 314 | GBBG | 2020 | -19.6 | 16.2 |
| 400 | GBBG | 2020 | -18.0 | 17.3 |
| 416 | GBBG | 2020 | -18.0 | 16.8 |
| 606 | GBBG | 2020 | -17.8 | 16.9 |
| 609M | GBBG | 2020 | -17.5 | 16.4 |
| 620 | GBBG | 2020 | -19.1 | 13.4 |
| 630 | GBBG | 2020 | -17.8 | 16.8 |
| 630M | GBBG | 2020 | -17.8 | 16.9 |
| 632 | GBBG | 2020 | -18.1 | 16.9 |
| UNK10 | GBBG | 2020 | -17.7 | 16.9 |
| UNK11 | GBBG | 2020 | -17.6 | 17.1 |
| UNK12 | GBBG | 2020 | -17.7 | 16.9 |
| UNK1 | GBBG | 2020 | -20.20 | 10.7 |
| 640 | GBBG | 2021 | -18.4 | 15.1 |
| 669 | GBBG | 2021 | -18.0 | 16.4 |
| 610 | GBBG | 2021 | -17.8 | 16.8 |
| 637 | GBBG | 2021 | -18.1 | 15.0 |
| 639 | GBBG | 2021 | -18.2 | 16.6 |
| 654 | GBBG | 2021 | -17.9 | 16.4 |
| 664 | GBBG | 2021 | -18.1 | 15.8 |
| 672 | GBBG | 2021 | -18.5 | 12.5 |
| 610M | GBBG | 2021 | -18.7 | 12.5 |
| 637M | GBBG | 2021 | -18.0 | 16.4 |
| UNK1 | GBBG | 2021 | -18.1 | 16.8 |
| UNK2 | GBBG | 2021 | -17.8 | 15.5 |
| UNK3 | GBBG | 2021 | -18.2 | 16.8 |
| UNK4 | GBBG | 2021 | -18.0 | 16.7 |
| UNK5 | GBBG | 2021 | -18.1 | 16.9 |
| UNK6 | GBBG | 2021 | -18.1 | 16.8 |
| UNK13 | GBBG | 2021 | -18.2 | 16.8 |

**Supplementary table S3**: All marine prey and urban refuse items included in stable isotope mixing models and associated isotopic values. Marine prey items were grouped *a priori* based on similar feeding habits and isotopic values. Fast-food meat and wheat were combined *a posteriori* into an “urban refuse” category and benthic and pelagic marine prey were combined *a posteriori* into a “marine prey” category.

| Diet item | Sample | n | ẟ^13^C ‰ | sd | ẟ^15^N ‰ | sd |
| --- | --- | --- | --- | --- | --- | --- |
| Fast-food meat | Chicken finger  Hamburger | 3  2 | -18.9 | ± 2.2 | 2.8 | ± 0.3 |
|  |  |  |  |  |  |  |
| Wheat-based food | White bread | 3 | -25.3 | ± 0.4 | 3.8 | ± 0.3 |
| Marine | Hard Clam (*Mercenaria mercenaria*) | 4 | -20.2 | ± 0.3 | 12.2 | ± 0.3 |
|  | Atlantic Menhaden (*Brevoortia tyrannus*) | 6 | -19.6 | ± 0.7 | 14.0 | ± 0.6 |
|  | Ribbed Mussel (*Geukensia demissa*)  Horseshoe Crab (*Limulus polyphemus*)  Spider Crab (*Libinia emarginata)*  Lady Crab (*Ovalipes ocellatus*)  Scup (*Stenotomus chrysops*) | 5  3  2  1  5 | -20.9  -18.3  -19.6  -18.0  -17.6 | ± 0.3  ± 0.2  ± 1.1  -  ± 0.7 | 12.4  16.5  15.6  14.5  16.1 | ± 0.3  ± 0.5  ± 0.6  -  ± 1.0 |

**Supplementary table S4**: Additional trophic enrichment factors (TEF) of dietary items collected from literature and associated estimated percentage of diet in great black-backed and herring gulls.

| Reference | Source | Diet item | TEF(^13^C)±sd | TEF(^15^N)±sd | GBBG estimated proportion of diet | HERG estimated proportion of diet |
| --- | --- | --- | --- | --- | --- | --- |
| Hobson et al. 1992^a^ | marine | marine | -0.3±0.8 | 3.1±0.2 | 93% ± 3 | 20% ± 4 |
|  | urban^b^ | wheat-based food | 1.2±0.6 | 2.2±0.2 | 7 % ± 3 | 80 % ± 4 |
|  |  | fast-food meat | 0.9±0.6 | 2.2±0.2 |  |  |
| Ogden et al.^c^ | marine | marine | 1.1±0.07 | 3.2±0.16 | 87% ± 5 | 15% ± 3 |
|  | urban | wheat-based food | 1.3±0.07 | 2.9±0.16 | 13% ± 5 | 85% ± 3 |
|  |  | fast-food meat | 1.3±0.07 | 2.9±0.16 |  |  |

^a^ Hobson et. al 1992 determined trophic enrichment factors for marine prey items in ring-billed gulls (*Larus delawarensis*).

^b^ TEFs for urban diet items were determined by feeding wheat-based turkey starter (here, representing wheat-based foods) and corn (here, representing fast-food meat) in Japanese quail (*Coturnix japonica*). No nitrogen TEF was documented in this study for corn, so we used the same TEF presented for wheat-based diet items.

^c^ Ogden et al. determined TEFs by feeding marine and terrestrial diet items to captive dunlins (*Calidris alpina*). Because the terrestrial diet items in Ogden et al. did not include corn-based diet items, we used TEFs for terrestrial sources to represent fast-food meat here.
